# Metabolic Checkpoints: Novel Avenues for Immunotherapy of Cancer

**DOI:** 10.3389/fimmu.2018.01816

**Published:** 2018-08-07

**Authors:** Ivan Shevchenko, Alexandr V. Bazhin

**Affiliations:** ^1^Institute for Immunology, Ludwig-Maximilians University, Munich, Germany; ^2^Department of General, Visceral and Transplantation Surgery, Ludwig-Maximilians University, Munich, Germany; ^3^German Cancer Consortium (DKTK), Partner Site Munich, Munich, Germany

**Keywords:** metabolic checkpoints, immune checkpoints, cancer immunotherapy, PD-1, checkpoint blockade, mammalian target of rapamycin pathway

## Abstract

Novel therapies targeting immune checkpoint molecules have redefined the treatment of cancer at advanced stages and brought hope to millions of patients worldwide. Monoclonal antibodies targeting immune-inhibitory receptors often lead to complete and objective responses as well as to durable progression-free survival where all other therapeutic approaches fail. Yet, many tumors show significant resistance to checkpoint blockade through mechanisms that are only starting to come to light. An alluring alternative strategy to reinvigorate anticancer immune responses comes from the emerging field of immuno-metabolism. Over the past few years, numerous studies revealed that many well-known metabolic playmakers also serve as critical checkpoints in immune homeostasis and immunity against tumors. Here, we survey recent insights into the intimate and intertwining links between T cell metabolic programs and environmental cues in the tumor milieu. Transferring these new findings from the bench to the bedside may soon entirely re-shape the field of cancer immunotherapy and significantly improve the lives of patients.

## Introduction

Immunotherapy has become a paradigm-shifting approach showing unmatched efficacy in patients with advanced malignancies. Targeting immune regulatory receptors, such as CTLA-4, PD-1, and PD-1 ligand (PD-L1), leads to spectacular clinical responses (1). Unfortunately, the success of these treatments is often limited to minor cohorts of patients. This calls for the development of alternative strategies for reinvigorating anticancer immunity. Recently, research into immuno-metabolism has emerged as an extraordinarily vibrant and productive area of study that is likely to become a launchpad for novel therapeutic approaches (2, 3). Over the past few years, the immunological community has witnessed a veritable Cambrian explosion of remarkable studies identifying the critical metabolic programs and checkpoints in the activation, differentiation, and migration of immune cells (2, 4). This research has illuminated the metabolic requirements for successful T cell-mediated effector responses and memory T cell generation in cancer. Furthermore, several groups have revealed the complex effects of multiple micro-environmental factors on T cell functionality in the tumor (5, 6). For instance, the pleiotropic roles of oxygen tension in the regulation of anticancer immunity are now coming to light.

Importantly, pathways controlling T cell responses to external challenges often converge on the same limited set of enzymes, transcription factors, and signaling complexes serving as metabolic checkpoints (7, 8). This highlights the elegant simplicity and dazzling complexity of T cell biology. Untangling these immuno-metabolic nodes will be essential for the rational design of future therapies for cancer.

## Immuno-Metabolic Checkpoints in T Cell Differentiation and Function

Metabolic changes occur throughout the lifespan of a T cell and provide the essential energetic currency and building blocks to help the T cell meet the emerging needs (4). Effective T cell responses against tumors strongly depend on the differentiation pathways taken up by individual CD4^+^ and CD8^+^ T cells upon their interaction with tumor antigens (9). The lineage commitment of stimulated T cells depends on the integration of the plethora of environmental cues and cell-intrinsic signals during activation, initial proliferation rounds, development of effector functions, and until final differentiation steps (2, 10).

Throughout their developmental path, T cells must strike a balance between increasing energy demands and a growing need for substrates to maintain their functionality and proliferation (2). Naive or quiescent T cells rely mainly on oxidative phosphorylation (OXPHOS), a highly efficient pathway for generating ATP from glucose. Upon activation, however, a switch to aerobic glycolysis takes place. In this rather inefficient process, only two ATP molecules are produced per each molecule of glucose. Switching to aerobic glycolysis might appear very inefficient, due to the low ATP/glucose ratio. Yet, at the same time, aerobic glycolysis yields a higher number of building blocks for anabolism. Importantly, the glycolytic switch is also essential for T cells to acquire diverse effector functions (e.g., production of IL-2, IFN-γ, etc.), since it relieves the blockade of IFN-γ mRNA translation by the glycolytic enzyme GAPDH (11).

The metabolic switch upon T cell activation is governed by a plethora of transcription factors and signaling pathways. Together, TCR engagement, costimulation, and cytokine signaling boost glycolysis by upregulating the expression of nutrient transporters (such as the Glut1 glucose importer) and activating the central metabolic regulator mammalian target of rapamycin (mTOR) complex (12–14). mTOR drives the development of all the effector T cell subsets but hampers peripheral Treg induction. This can be accounted for the requirement of intensified glycolysis during effector T cell expansion, while Treg cells primarily deploy OXPHOS and oxidation of fatty acids. mTOR comprises two distinct complexes, mTORC1 and mTORC2, and orchestrates cellular responses to changes in nutrient levels and energy status (15). Costimulation *via* CD28 activates PI3K recruiting 3-phosphoinositide-dependent protein kinase-1 and Akt, which, in turn, activates mTOR. This pathway leads to the quick upregulation of Glut1 expression and to its increased transport to the plasma membrane. The rapid intensification of glucose import is critical for efficient T cell activation, clonal expansion, and survival.

Of note, a study by Macintyre et al. (16) suggested that Glut1 is only indispensable for the differentiation of Th1, Th2, and Th17 cells, but not CD8^+^ T cells or Tregs. A plausible explanation might be coming from recent proteomic studies indicating that Glut1 and Glut3 protein levels are comparable in CD8^+^ T cells (17). Therefore, glucose transporters can act in a somewhat redundant fashion to meet the demands of T cells for glucose.

The multifaceted role of glucose uptake in Treg biology has recently been further elucidated in a study by Rathmell and colleagues who identified toll-like receptor (TLR) signals that drive Treg cell proliferation *via* PI(3)K–Akt–mTORC1 signaling, which intensifies glycolysis and glucose import by Glut1 (18). Conversely, TLR-induced mTORC1 signaling also diminished the ability of Tregs to suppress effector T cell proliferation. In line with previous studies, the transcription factor Foxp3 dampened the effects of PI(3)K–Akt–mTORC1 signaling to hamper glycolysis and anabolism while boosting OXPHOS and catabolic pathways. Likewise, although Glut1 expression promoted Treg expansion, it also reduced their suppressive activity and Foxp3 expression. This indicates that Treg cells might occasionally switch to aerobic glycolysis and expand when they receive inflammatory signals, and subsequently switch back to FAO and OXPHOS to achieve maximal suppressive potency. These findings further highlight glycolysis as a critical metabolic axis in maintaining the immunological balance. Therefore, glycolytic enzymes and nutrient transporters represent attractive targets for future immuno-metabolic therapies of cancer. Specifically, administration of glucose uptake inhibitors or Glut knockdown in adoptively transferred cells could be instrumental for pushing antitumor T cell differentiation toward long-lived memory cell lineage for the generation of enduring antitumor immunity (9, 19).

Migration of activated Treg cells to the site of inflammation is crucial for their immune-inhibitory function (20). Kishore et al. (21) have studied the metabolic needs for migratory Treg. They demonstrated that glycolysis strongly promotes Treg migration and is triggered by a PI3K–mTORC2-mediated pathway driving the activation of the enzyme glucokinase. These findings also suggest a new attractive strategy to avert Treg infiltration into tumors by manipulating their metabolic programs.

The transcription factors c-Myc and hypoxia-inducible factor-1α (HIF-1α) coordinately activate the genes required for the vigorous proliferation of effector T cells during clonal expansion (22, 23). Importantly, both Myc and HIF-1α are under the control of the mTOR complex. C-Myc promotes the expression of enzymes involved in aerobic glycolysis and glutaminolysis and fine-tunes these metabolic pathways to the biosynthesis of lipids, amino acids, and nucleic acids. HIF-1α mediates T cell responses to oxygen levels and also promotes glucose uptake and breakdown (24). Thus, the same transcription master regulators that control such fundamental processes as cell proliferation and cellular response to oxygen tension are also responsible for adjusting T cell metabolism to emerging needs. This underscores the startling universality and efficiency of these most fundamental mechanisms of epigenetic regulation and indicates that a robust reprogramming of antitumor T cell metabolism might be achieved by only targeting a few select molecules.

The dichotomy between glycolysis versus OXPHOS and FAO is not only central to the control of T cell activation and effector function but is also decisive in the fate of differentiating T cells. Most importantly, in precursors of long-lived memory cells, OXPHOS and FAO are engaged to balance out the effects of aerobic glycolysis (25). A pivotal role for mitochondria (26) in these cells is manifest in the dynamics of their ultrastructure. In memory T cells, mitochondrial cristae fuse into elaborate networks, while mitochondria in effector T cells exhibit extensive fission. The functionality of mature memory cells is also sustained by the higher biomass and spare respiratory capacity of their mitochondria through IL-15-driven upregulation of carnitine palmitoyl-transferase. This enzyme drives FAO and engenders stronger and more protracted OXPHOS and glycolysis upon restimulation.

Importantly, a wealth of recent evidence illuminates the possibilities to improve clinical responses to immune checkpoint inhibitors by combining these therapies with modulation of metabolic pathways. For instance, a series of elegant studies by Chi and colleagues (7, 27, 28) have demonstrated the intimate interplay between immune and metabolic checkpoints in T cell differentiation. Specifically, mTORC1 signaling was established as a key “rheostat” in Treg cell function (28). Treg-intrinsic disruption of mTORC1 led to a drastic slump in Treg suppressive activity and launched a deadly early-onset inflammatory disease. Raptor/mTORC1 signaling in Tregs boosted the metabolism of cholesterol and lipids, while the mevalonate pathway proved essential for orchestrating Treg proliferation and upregulated expression of the checkpoint molecules CTLA4 and ICOS. Another study by the same group highlighted the role of autophagy in the lineage stability and survival of Treg cells (29). Treg cell-specific deficiency in Atg7 or Atg5, two pivotal genes in autophagy, resulted in a diminished Treg compartment, improved antitumor immunity, and development of inflammatory disorders. Autophagy kept in check mTORC1, c-Myc, and glycolytic enzymes, thereby coupling environmental signals to metabolic homeostasis.

## Metabolic Competition in the Tumor Microenvironment

**Figure 1:**
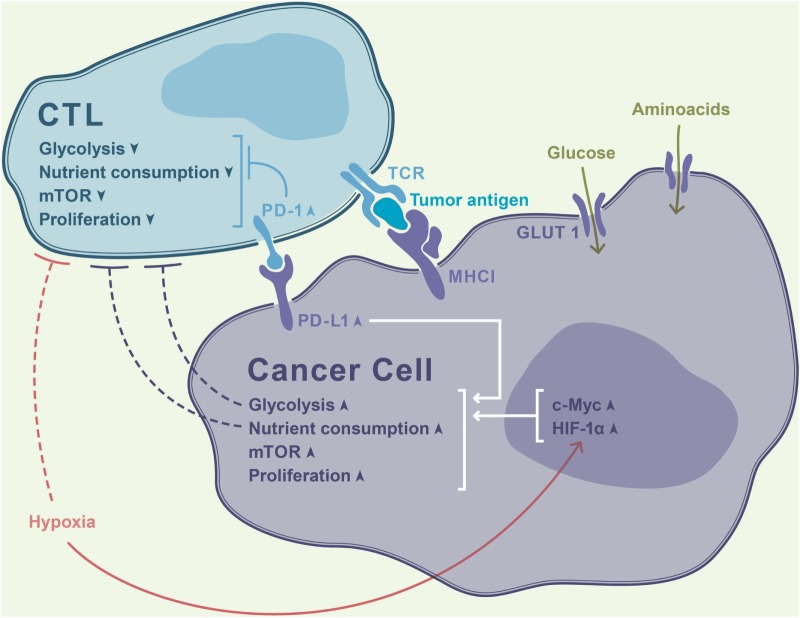
Rapidly proliferating tumor cells avidly consume nutrients from the microenvironment, thereby outcompeting T cells in a contest for metabolic fitness. Increased activity of hypoxia-inducible factor-1α (HIF-1α) driven by tumor hypoxia further intensifies glycolysis and exacerbates the paucity of glucose in the milieu. This results in T cell deprivation of essential fuel for their effector function, resulting in a thwarted antitumor immune response. PD-1 signaling in T cells dampens their glycolytic activity and proliferation, while PD-1 ligand (PD-L1) ligation leads to opposite effects in cancer cells.

Recent studies have directly linked T cell metabolism, T cell exhaustion, and antitumor immunity. In the tumor, the scarcity of nutrients can profoundly affect cell proliferation, survival, and functionality. T cell-infiltrating tumors become enmeshed into teeming metabolic networks established within the hostile microenvironment and are forced to face ruthless competition for nutrients. Cancer cells can express various enzymes that deprive T cells of critical substrates and produce immune-inhibitory metabolites (Figure 1). For instance, many tumors contain large amounts of indoleamine 2,3-dioxygenase (IDO), an enzyme that eliminates tryptophan from the microenvironment and hampers T cell proliferation (30). Of note, despite the significant efficacy of IDO inhibitors in mouse models of cancer, these compounds have shown no measurable antitumor efficacy in clinical settings (31). The potential advantages and pitfalls of therapeutically exploiting the metabolic differences between normal and malignant cells have recently been comprehensively surveyed by Martinez-Outschoorn and coauthors (32).

In two pioneering studies, published side by side in *Cell* (5, 6), the groups of Susan Kaech and Erika Pearce reported that tumor cells outcompete T cells for glucose, thereby dampening their effector function and evading immune destruction. Ho et al. (5) demonstrated that unperturbed glucose metabolism in T cells is critical for TCR-induced Ca^2+^ flux. Extracellular glucose promoted accumulation of phosphoenolpyruvate, a glycolytic metabolite that inhibited sequestering of Ca^2+^ from the cytoplasm into the ER, thereby sustaining activation-induced Ca^2+^ flux and T cell effector function. Conversely, increased expression of the glycolysis gatekeeper hexokinase 2 in tumor cells facilitated tumor escape from CD4^+^ T cell-mediated immune surveillance, further corroborating the importance of metabolic competition between tumor-infiltrating lymphocytes (TILs) and tumor cells.

In the other study, Chang et al. (6) demonstrated that signaling through PD-L1 in tumor cells promotes glycolysis *via* activation of the AKT/mTOR pathway. Therapeutic blockade of PD-L1 decreased glycolysis rate by triggering PD-L1 internalization, restored glucose levels in the microenvironment, and hindered tumor progression. These data provided a breakthrough in our understanding of the deeply intertwined metabolic and immune checkpoint signaling pathways and underscore the potential of future immune-metabolic therapies.

Conversely, rather than targeting the metabolism of tumor cells, Ho et al. (5) put forward an alternative strategy to reinforce T cell function by artificially increasing PEP levels in adoptively transferred tumor-reactive T cells. PEP carboxykinase (PCK1) catalyzes conversion of oxaloacetate into PEP. Overexpression of PCK1 in transferred T cells allowed the authors to restore TCR-driven Ca^2+^ flux and anticancer T cell activity, thus overriding the effects of low glucose levels in the tumor microenvironment. Overall, these reports provided inspiring examples of reprogramming T cell metabolism to enhance the efficacy of adoptive cell therapies for cancer and spawned many further studies exploring T cell metabolic dysfunction in the tumor.

For instance, the latest works by Delgoffe and colleagues shed new light on various aspects of T cell metabolism in cancer. For example, Scharping et al. (33) reported a continuous loss of mitochondrial mass and functionality in tumor-infiltrating T cells, which proved restricted to the tumor milieu and was not a mere consequence of activation. Due to chronic Akt signaling, TILs showed dwindling expression of PPAR-gamma coactivator 1α (PGC1α), a vital factor in mitochondrial biogenesis. Reprogramming TILs through forced expression of PGC1α reinvigorated their metabolic and effector function. Interestingly, in a study by Wherry and colleagues (34), PD-1 was shown to inhibit PGC1α and thereby serve as a dominant-negative regulator of glycolysis in activated T cells.

More recently, it has been reported that T cell activation leads to a rapid glycolytic switch independently of transcription, translation, costimulation through CD28, or Akt signaling and without an increase in glucose uptake or in the activity of glycolytic enzymes (35). Instead, TCR engagement enhances activation of pyruvate dehydrogenase kinase 1 (PDHK1), thereby hampering import of pyruvate into mitochondria and promoting its cleavage into lactate. Intriguingly, inhibiting PDHK1 revealed that the early glycolytic switch is required for immediate cytokine production but not for cytotoxicity. Recently, Menk et al. (36) demonstrated that ligation of 4-1BB (a costimulatory molecule highly expressed on exhausted T cells) provides metabolic support to tumor-infiltrating T cells by enhancing their mitochondrial capacity and engaging PGC1α-mediated pathways. Remarkably, 4-1BB stimulation combined with PD-1 blockade resulted in robust antitumor immunity. This study further highlights the potential of combinatorial strategies targeting immune-metabolic checkpoints for reshaping the immune-inhibitory tumor milieu.

## Oxygen Sensing

The transcription factor HIF-1α is a critical driver of effector T cell responses, which is particularly important for the differentiation of CD4^+^ Th17 cells (37), CD8^+^ T cell effector function (22), and interferon IFN-γ production by T regulatory cells. Besides governing the glycolytic switch in Th17 cells, HIF-1α also transactivates the gene encoding the Th17 master transcription factor RORγt (37). It has been demonstrated that HIF-1α-mediated T cell response to hypoxia upregulates the expression of Glut1, as well as glycolytic enzymes, and therefore strongly affects T cell differentiation and function. For instance, Cretenet et al. (38) demonstrated that hypoxia significantly enhances Glut1 upregulation in response to TCR stimulation. Furthermore, Glut1^hi^ T lymphocytes displayed more pronounced Th1 effector phenotype and higher proliferation rate than their Glut1^lo^ counterparts, both under normoxic and hypoxic conditions. Therefore, enhancing glucose uptake in adoptively transferred T cells might allow for efficiently countering hypoxia-driven immune suppression.

The activity of HIF-1a is tightly controlled by the oxygen-sensing prolyl-hydroxylase (PHD) proteins. Under normoxia, PHDs hydroxylate HIF-1α and HIF-2α, thereby targeting them for degradation. In their recent study, Restifo and colleagues (39) found that expression of PHDs in T cells ensures local tolerance for harmless antigens in the lung but markedly facilitates the seeding of circulating tumor cells. In line with their role as HIF-1α inhibitors, PHDs limited pulmonary Th1 responses, promoted Treg cell induction, and dampened CD8^+^ T cell function. Importantly, it was shown that the effects of the PHD enzymes are primarily mediated by the repression of HIF-driven glycolytic metabolism.

Specifically, T cells stimulated in TGF-β-containing media exhibited a PHD-dependent reduction of glycolytic activity. On the other hand, PHD-deficient CD4^+^ T cells exhibited accelerated uptake of glucose and switched to an anaerobic metabolic program. PHD proteins also restrained glycolysis in CD8^+^ T cells. Remarkably, targeting mTOR-driven glycolytic metabolism with rapamycin and 2-deoxyglucose completely blocked spontaneous Th1 development and partially restored iTreg cell differentiation in PHD-deficient T cells. Hence, oxygen sensing appears to coordinate transcriptional and metabolic programs driving the differentiation of Th1 and iTreg cells. Recent clinical studies have demonstrated the efficacy and overall safety of PHD inhibitors in patients with anemia and other hypoxia-driven pathologies (40). However, the complexity and the near-universal nature of the HIF pathways necessitate thorough evaluation of adverse effects. Collectively, targeting PHD proteins and other links in oxygen sensing is an alluring strategy to tilt the balance between immune activation and immune suppression in the tumor.

## Conclusion and Future Directions

Metabolism is integral to every biological process. The immune system largely consists of mobile cells that patrol the body and need to adapt to diverse challenging environments. This requires tight and sophisticated coordination of their bioenergetic machinery with their homeostatic pathways and effector functions. Here, we summarized the latest studies shedding light onto the specific roles of particular substrates, enzymes, and metabolic regulators in T cell differentiation and antitumor activity. Despite the startling progress that has been made in the field within just a few years, several critical questions remain unanswered. For instance, the timing and the mutual causality of the major metabolic switches during T cell differentiation remain largely elusive. Specifically, it will be crucial to capture the exact moments when differentiating T cells pass through a given metabolic checkpoint and how this affects the identity and the fate of a T cell. Likewise, further research is necessary to gain a more integrative view of how intratumoral T cells are affected by hypoxia and relentless metabolic competition imposed by cancer cells.

In the same vein, there is a pressing need to assess the therapeutic efficacy of a broader spectrum of genetic modifications targeting diverse metabolic regulators and oxygen sensors in adoptively transferred T cells. Importantly, the ubiquity of many metabolic pathways calls for careful target selection and cautious design of therapeutic regimens based on highly specific small molecule inhibitors. Overall, future immune-metabolic therapies have all the potential to make a critical difference for patients suffering from otherwise untreatable cancers.

## Author Contributions

IS and AB wrote this manuscript. IS generated the figures.

## Conflict of Interest Statement

The authors declare that the research was conducted in the absence of any commercial or financial relationships that could be construed as a potential conflict of interest.
